# DAB2IP predicts treatment response and prognosis of ESCC patients and modulates its radiosensitivity through enhancing IR-induced activation of the ASK1-JNK pathway

**DOI:** 10.1186/s12935-022-02535-9

**Published:** 2022-03-05

**Authors:** Zhuting Tong, Weiyang Fang, Meng Xu, YeYe Xia, Rui Wang, Yue Li, Tianqi Zha, Liang Xiao, Shuhao Pan, Huiping Chai, Lei Zhao, Hao Wang, Huaguang Pan, Xiangcun Chen

**Affiliations:** 1 Department of Radiation Oncology, The First Affiliated Hospital of Anhui Medical University, Hefei, China; 2 Department of Electrocardiography, The First Affiliated Hospital of Anhui Medical University, Hefei, China; 3 Department of Oncology, Changhai Hospital, The Second Military Medical University, Shanghai, China; 4 Guangdong Institute of Gastroenterology, The Sixth Affiliated Hospital of Sun Yat-Sen University, Sun Yat-Sen University, Guangzhou, China; 5 Department of Thoracic Surgery, The First Affiliated Hospital of Anhui Medical University, Hefei, China; 6 Department of Radiation Oncology, Sun Yat-Sen University Cancer Center, Guangzhou, China

**Keywords:** Esophageal squamous cell carcinoma, DAB2IP, Chemoradiosensitivity, ASK1, JNK

## Abstract

**Background:**

Disabled homolog 2 interacting protein (DAB2IP) plays a tumor-suppressive role in several types of human cancers. However, the molecular status and function of the DAB2IP gene in esophageal squamous cell carcinoma (ESCC) patients who received definitive chemoradiotherapy is rarely reported.

**Methods:**

We examined the expression dynamics of DAB2IP by immunohistochemistry (IHC) in 140 ESCC patients treated with definitive chemoradiotherapy. A series of in vivo and in vitro experiments were performed to elucidate the effect of DAB2IP on the chemoradiotherapy (CRT) response and its underlying mechanisms in ESCC.

**Results:**

Decreased expression of DAB2IP in ESCCs correlated positively with ESCC resistance to CRT and was a strong and independent predictor for short disease-specific survival (DSS) of ESCC patients. Furthermore, the therapeutic sensitivity of CRT was substantially increased by ectopic overexpression of DAB2IP in ESCC cells. In addition, knockdown of DAB2IP dramatically enhanced resistance to CRT in ESCC. Finally, we demonstrated that DAB2IP regulates ESCC cell radiosensitivity through enhancing ionizing radiation (IR)-induced activation of the ASK1-JNK signaling pathway.

**Conclusions:**

Our data highlight the molecular etiology and clinical significance of DAB2IP in ESCC, which may represent a new therapeutic strategy to improve therapy and survival for ESCC patients.

## Introduction

Esophageal carcinoma is the eighth most common cancer and the sixth leading cause of cancer-related deaths worldwide. Esophageal squamous cell carcinoma (ESCC) is the dominant histological type of esophageal cancer in China, with a 5-year overall survival less than 30% [1]. At present, definitive concurrent chemoradiotherapy (CRT) has been established as the standard treatment for advanced localized ESCC [2]. However, therapeutic outcomes are not satisfactory, even with the recent rapid development of diagnostics and therapies. Chemoradioresistance is one of the most important factors for the poor prognosis in ESCC patients [3]. Currently, the molecular mechanisms underlying chemoradioresistance in ESCC have not been clearly elucidated. Thus, it is necessary to identify novel markers that can predict responses to chemoradiotherapy, so as to find novel therapeutic targets and develop new modalities of treatment.

DAB2IP is a novel member of the Ras-GTPase-activating protein family [4]. *DAB2IP* has been identified as an EZH2 target gene by epigenetic silencing mechanism. The N-terminal domain of DAB2IP interacts with tumor suppressor DOC2/DAB2 to form a unique protein complex that has negative regulatory activity targeting the Ras-mediated signaling pathway [5]. Recently, there has been increasing evidence that reduced expression of DAB2IP occurs in several types of human malignancies, including prostate cancer [6], lung cancer [7], hepatocellular carcinoma [8], renal cancer [9], and breast cancer [10]. Loss expression of DAB2IP was observed to correlate closely with tumor aggressiveness and/or poor patient prognosis. Particularly, downregulation of DAB2IP expression was reported to be associated with resistance to chemo or radio therapy and worse outcome in several types of human cancers [6, 11–16]. Additionally, Sun et al. reported that decreased expression of DAB2IP correlated with ESCC progression and poor prognosis in surgically resected samples [17]. To date, however, the molecular status of the DAB2IP gene in ESCC patients treated with definitive chemoradiotherapy and the relationship between the expression of DAB2IP and chemoradiosensitivity in ESCC has not been elucidated.

Notably, our group’s previous work demonstrated that EZH2, an upstream inhibitory regulatory protein of DAB2IP, serves as a promising diagnostic biomarker for hepatocellular carcinoma [18] and supports nasopharyngeal carcinoma cell aggressiveness [19]. Most importantly, we further revealed that high expression of EZH2 was correlated with a poor response to chemoradiotherapy in ESCC patients [20]. These evidences promote us to feel great interest in determining the significance of DAB2IP, as an identified repressive target gene of EZH2, in CRT sensitivity and its effect on the prognosis of ESCC patients treated with definite CRT.

In this study, to determine whether abnormalities of DAB2IP were involved in the pathogenesis and chemoradiosensitivity of ESCC, we applied immunohistochemistry (IHC) analysis to examine the expression of DAB2IP in a cohort of biopsy specimens of primary ESCC patients treated with definitive CRT. Furthermore, to examine whether the expression of DAB2IP could predict the ESCC response to CRT and patient clinical outcomes, we evaluated the correlation between DAB2IP expression and patient clinical/prognostic factors. In addition, the underlying molecular mechanisms of DAB2IP involved in chemo/radioresistance in ESCC were investigated.

## Materials and methods

### Cell culture

The three ESCC cell lines (i.e., TE1, EC109, and Kyse150) were purchased in 2015 from the Cell Bank of the Chinese Academy of Sciences (Shanghai, China) where they were characterized by mycoplasma detection, DNA-Fingerprinting, isozyme detection and cell vitality detection. The ESCC cell line Kyse30 was kindly provided by Prof. Xiaofeng Zhu (Sun Yat-Sen University Cancer Center) in 2014. All cell lines were maintained in RPMI-1640 supplemented (11875093, Gibco, Thermo Fisher Scientific, MA, USA) with 10% fetal bovine serum (FBS) (10100147, Gibco, Thermo Fisher Scientific, MA, USA) and were authenticated 3 months before the beginning of the study (2015) based on viability, recovery, growth and morphology by the supplier. All the cell lines have not been in culture for greater than 2 months.

### Antibodies and chemical reagents

Primary antibodies used in this study: rabbit anti-DAB2IP (ab87811, Abcam, UK); rabbit anti-cleaved poly (ADP-ribose) polymerase (PARP, Asp214), rabbit anti-cleaved caspase-3 (Asp175), mouse anti-Phospho-Histone H2AX (γ-H2AX) (Ser139), rabbit anti-53BP1, rabbit anti-AKT and anti-Phospho-AKT (Ser473), rabbit anti-JNK and anti-Phospho-JNK, rabbit anti-ERK1/2 and anti-Phospho-ERK1/2, rabbit anti-ASK1 and anti-Phospho-ASK1 (ser966), rabbit anti-14-3-3, rabbit anti-Thioredoxin and α-tubulin (Cell Signaling Technology, Boston. MA). Secondary antibodies: Dylight 549-conjugated goat anti-rabbit IgG (Proteintech Group, Inc., Chicago, IL), Alexa Fluor 488-conjugated goat anti-mouse IgG (Invitrogen, Carlsbad, CA). Peroxidase-conjugated goat anti-rabbit IgG and goat anti-mouse IgG (Santa Cruz Biotechnology, Santa Cruz, CA). Annexin V-FITC/PI apoptosis detection kit (Vazyme Biotech, China).

### Ethics approval and consent to participate

All patients authorized the use of their specimens by written informed consent. This study was approved by the Institute Research Medical Ethics Committee of Anhui Medical University (Hefei, China). This study was performed in accordance with the Declaration of Helsinki.

### ESCC patients

In this study, a total of 140 ESCC patients treated with definitive CRT were consecutively selected from The First Affiliated Hospital of Anhui Medical University (Hefei, China) and Cancer Center of Sun Yat-Sen University (Guangzhou, China) between March 2007 and August 2015. In addition, 25 samples of normal esophageal mucosa were used for controls. The cases selected were based on the following criteria: (a) all the cases have reliable biopsy specimens and follow-up date; (b) patients without previous treatment, malignant disease or a second primary tumor; (c) no previous treatment or severe complications; (d) no distant metastases except for supraclavicular or celiac lymph nodes; (e) Karnofsky ≥ 70; (f) all the samples were endoscopic biopsy specimens obtained before CRT. 14 pairs of cancer tissues and adjacent normal esophageal specimens were snap-frozen in chilled liquid nitrogen and stored at − 80 °C until further processing. The study was approved by the medical ethics committee of our institutes.

### Chemoradiotherapy

All the 140 patients received the same concurrent chemoradiotherapy with PF (Cisplatin/5-fluorouracil) regimen. Cisplatin was administered as i.v. drip at a dose of 60–80 mg/m^2^ on days 1 to 2; 5-fluorouracil 3000 mg/m^2^ was administered as a continuous i.v. infusion for 48 h on days 1–2. Two cycles of chemotherapy were done during radiotherapy at 4-week intervals.

Radiotherapy was performed by three-dimensional conformal radiotherapy or intensity-modulated radiotherapy with 6 MV X-ray. The gross tumor volume (GTV) was defined as the primary tumor and positive lymph nodes. The clinical target volume (CTV) included the GTV with a 3–4 cm margin in the cephalad and caudal directions, and a radial margin of 0.5–1.0 cm. CTV also comprised the regional lymphatic regions. The planning target volume (PTV) included the CTV with a uniform 0.8 cm expansion margin in all directions. A total prescription dose of 60–70 Gy was delivered in 1.8–2.0 Gy fractions over 6–7 weeks.

### Clinical response evaluation and follow-up

The response to CRT was evaluated clinically for primary lesions based on esophagography, endoscopy and CT 2 months after CRT according to the following criteria. Complete response (CR) was defined as no evidence of disease on imaging and complete resolution of all assessable lesions by endoscopic biopsy. Partial response (PR) was defined as a 30% or greater reduction in tumor maximum dimension and no progression of assessable lesions. Stable disease (SD) was defined by a reduction by < 30% or increase of < 20% in tumor size. All these conditions had to last for at least 4 weeks and there was no appearance of new lesions. Progressive disease (PD) was defined as an increase ≥ 20% in tumor size or the appearance of new lesions. We divided these categories into two groups: CR and non-CR (PR/SD/PD).

The patients were followed every 3 months for the first year and then every 6 months for the next 2 years and finally annually. The diagnostic examinations consisted of esophagography, CT, chest X-ray, abdominal ultrasonography and bone scan when necessary to detect recurrence and/or metastasis. The disease-specific survival (DSS) was defined as the time from diagnosis to the date of cancer-related death or when censured at the latest date if patients were still alive.

### Western blotting

Equal amounts of whole tissue or cell lysates were resolved by SDS-polyacrylamide gel electrophoresis (PAGE) and transferred on a polyvinylidene difluoride (PVDF) membrane (Millipore, Bed-ford, MA, USA) followed by incubating with primary antibodies against DAB2IP, cleaved-PARP, cleaved-caspase3, AKT, p-AKT, JNK, p-JNK, p-ERK1/2, and ERK1/2, Phospho-ASK1(ser966), ASK1, 14-3-3, Trx, and α-tubulin, respectively. The immunoreactive proteins were detected with enhanced chemiluminescence detection reagents (Thermo Pierce, Cramlington, UK) according to the manufacturer’s instructions. Bicinchoninic acid (BCA) assay was performed to determine protein concentrations.

### Immunohistochemistry (IHC) staining

IHC analysis was performed to examine DAB2IP expression levels in ESCC specimens. The staining protocol used in this study has been described previously [21]. DAB2IP antibody (1:200 dilution, Abcam) was used in IHC staining. In our study, we divided the cell staining percentage into 10-scale system ranging from 0 to 9; a score of 0 indicated staining was < 10%, a score of 1 indicated staining was > 10% but ≤ 20%, a score of 2 indicated staining was > 20% but ≤ 30%, and so forth. Meanwhile we divided the staining intensity into 4 degrees, ranging from 0 to 3, 0 indicated negative, 1 indicated weak intensity, 2 indicated moderate intensity, and 3 indicated strong intensity.

The two individual parameters were added, resulting with total score ranging from 0 to 12. The stained tissue sections were reviewed and scored separately by two pathologists blinded to the clinical parameters. The concordance rate of 88.9% among the evaluated results from the pathologists demonstrated that this scoring method was highly reproducible. The value was selected until at least two pathologists reported consistent results. In cases where scoring was completely different, pathologists worked to reach a consensus on the score.

### X-tile

Camp et al. [22] previously developed a graphical method, named X-tile plot, which can present a new tool for the evaluation of biological relationships and discover cut-points based on marker expression. The X-tile plots allows the determination of an optimal cutoff score while correcting for the use of minimum P statistics by Miller-Siegmund P-value correction. According to the X-tile plots, we categorized the samples into low (IHC score ≤ 5) and high (IHC score > 5) expression subgroups based on a cut-point determined by X-tile software related to survival status.

### Knocking down of DAB2IP by lentiviral short hairpin RNA (shRNA)

The packaging protocol of shRNA lentivirus was performed as described previously [23] with slight modification. Briefly, the vector pLLU2G (kindly gifted by Professor Peng Xiang, Center for Stem Cell Biology and Tissue Engineering, Sun Yat-Sen University) used in this study was derived from pLL3.7 and contained separate green fluorescent protein (GFP) and short hairpin RNA (shRNA) expression elements, as well as elements required for lentiviral packaging. The target sequences of DAB2IP and control Renilla luciferase (luc) for constructing lentiviral shRNA is 5′-GTAATGTAACTATCTCACCTA-3′ [24] and 5′-GTAGCGCGGTGTATTATAC-3′ respectively. The ASK1 shRNA lentiviral particles were obtained from Santa Cruz Biotech (sc-29748-V). Packaging of viruses was performed by transient transfection of 293FT cells with a transfer plasmid and three packaging vectors: pMDLg/pRRE, pRSV-REV and pCMV-VSVG. Seventy-two hours after transfection, the lentiviral particles were collected and filtered, then concentrated by ultra centrifugation at 50000×*g* for 2.5 h at 4 °C. Subsequently, we infected the ESCC cancer cell lines with the lentivirus in a 6-well plate. Four days after infection, the knockdown efficiency of DAB2IP or ASK1 was examined by Western blotting.

### Mammalian expression plasmids construction and transfection

DAB2IP fragment was amplified by PCR and cloned into pcDNA3.1 plasmid. Cells were transfected with pcDNA3.1-DAB2IP or the control plasmid pcDNA3.1 (+) using Lipofectamine 3000 transfection reagent (Thermo Fisher Scientific, Waltham, MA, USA) according to the manufacturer’s instructions. For the establishment of DAB2IP stably expressed Kyse150 ESCC cells, 48 h after transfection, the cells were split at a ratio of 1:10, and subsequently maintained in selective medium containing 250 μg/ml of G418 (Invitrogen). After 6 weeks of selection, resistant colonies stably transfected with pcDNA3.1-DAB2IP or pcDNA3.1 (+) were pooled for further experiments.

### Determination of half maximal inhibitory concentration, IC50

Briefly, cells were seeded in 96-well plates in 3 replicate wells and treated with multiple incremental doses of cisplatin. Cell viability was determined by A Queous One Solution MTS kit (Promega) according to the manufacturer’s instructions, and the absorption was read at 490 nm. Curves were fitted using nonlinear regression in the GraphPad Prism 5.0 (Graph Pad Software Inc., San Diego, CA, USA) and IC50 was calculated. Data was presented as means ± standard deviation (SD) from three independent experiments.

### Annexin V-fluorescein isothiocyanate (FITC)/propidium iodide (PI) apoptosis detected by flow cytometry

To quantify the apoptotic cells, the occurrence of apoptosis was determined by staining cells with both Annexin V-FITC and PI. The apoptosis assay was conducted using the protocol according to manufacturer’s instructions (Vazyme Biotech, China). Each sample was then subjected to analyses by flow cytometry (BD Biosciences, San Jose, CA, USA).

### Clonogenic assays after radiation and Linear-Quadratic Model

Kyse150-DAB2IP, EC109-shDAB2IP and their corresponding control cells (Kyse150-vector, EC109-shluc) were seeded in 60 mm dishes with different cell numbers for each dose group (500–8000 cell/dose). At 24 h after seeding, cells were subjected to radiation (0, 2, 4, 6, 8, or 10 Gy) using a linear accelerator with 6 MV photons (Varian Associates Inc., Palo Alto, California). The field size was 30 × 30 cm^2^ and source surface distance was 100 cm. Then the cells were incubated in complete medium to allow colony growth for a period of 14–21 days. The surviving colonies (> 50 cells/colony) were stained with Giemsa (Invitrogen) and manually counted. Data from radiation exposure cells were normalized to the untreated cells (scored as 100% colony forming ability). Plating efficiencies and survival fractions were calculated to obtain survival parameters and plot cell survival curves. All experiments were performed in triplicate and data were presented as means ± SD. The radiation survival curves were fitted according to the linear quadratic (LQ) model by GraphPad Prism 5.0 (GraphPad, SanDiego, CA): as Survival Fraction (SF) = exp (− αD − βD^2^). We calculated the Sensitization Enhancement Ratio (SER) as follows [25]: SER = SF2 of experimental cells/SF2 of the corresponding control cells. SF2 means the surviving fraction at 2 Gy (SF2).

### Immunofluorescence

Cells were grown on glass coverslips up to 80% confluency. Then, cells were washed twice with PBS, fixed in 4% paraformaldehyde and processed for immunofluorescence staining. Frozen tissues were embedded in OCT and sectioned, then mounted on glass slides. The mounted tissues were air dried overnight fixed in 4% paraformaldehyde. The cell climbing slices and frozen tissue section were double immunostained with mouse anti-human antibody phospho-Histone H2AX (γ-H2AX) and rabbit anti-human 53BP1 antibody in a humidified chamber overnight at 4 °C. Cells were washed with PBS twice and primary antibodies were visualized by DyLight 549 conjugated Goat anti-Mouse IgG and Alexa Fluor 488 conjugated Goat anti-Rabbit IgG. DNA were stained with DAPI. Images were collected on the Olympus FluoView confocal microscopes and analyzed with FV10-ASW viewer software (Olympus, Tokyo, Japan). The number of 53BP1 and γ-H2AX fusion co-positive (yellow) foci per nucleus was calculated according to the formula $\sum_{{{\text{foci}}}} /{\text{n}}_{{{\text{total}}\;{\text{cells}}}} .$ The $\sum_{{{\text{foci}}}}$ means all the foci recorded in the cells counted at a given time point and the n_total cells_ represents the total number of cells assessed at that time point. All counting was performed manually by a single operator to minimize variation and then statistical analyzed. All experiments were repeated at least three times.

### In vivo experiments

We used 6- to 8-week-old female BALB/c nude mice. Kyse150-vector and Kyse150-DAB2IP cells at the concentration of 1 × 10^6^ per site were subcutaneously injected into the right flanks of mice. When xenograft tumors had grown to the volume of 180 mm^3^, tumors were treated with 6 Gy of radiation. Tumor diameters were measured every 3 days by calipers, and tumor volumes were calculated using the formula (length × width^2^/2). After 33 days, all mice were euthanized by intraperitoneal injection of pentobarbital (200 mg/kg). All experimental procedures were approved by the Institutional Animal Care and Use Committee.

### Immunoprecipitation (IP)

Cells were lysed by lysis buffer (50 mM Tris, pH 7.5, 150 mM NaCl, 1 mM EDTA, 1% Triton X-100) containing 1 mM sodium orthovanadate (New England Biolabs, Beverly, MA) plus a mixture of protease inhibitor cocktail (Sigma) for 10 min on ice, then sonicated and the cell debris was removed by centrifugation (18,000×*g* for 30 min). Bicinchoninic acid (BCA, Pierce, Rockford, IL) assay was preformed to determined protein concentrations. Cells lysates was precleared by incubation with Protein A/G beads (Sigma) for 1 h at 4 °C to eliminate nonspecific binding. The lysates were then subjected to inmmunoprecipitation overnight at 4 °C with indicated antibodies or purified mouse/rabbit immunoglobulin G (Sigma), followed by incubation with protein A/G beads for 2 h. Precipitates were washed 4 times with a lysis buffer before resuspending with a 2× protein sample buffer, and were boiled for 5 min to release the bound proteins. Subsequently, the proteins were analyzed by Western blot analysis with the indicated antibodies.

### Statistical analysis

Statistical analysis was performed with SPSS software (SPSS Standard version 23.0, SPSS, Chicago, IL). The Chi-square test was used to evaluate the relationship between DAB2IP expression and clinicopathological variables. Pearson contingency coefficient (r_p_) was used to analyze the relationship between DAB2IP expression and CRT response. Survival curves were plotted by the Kaplan–Meier method and compared by the log-rank test. Multivariate survival analysis was performed on all parameters that were found to be significant on univariate analysis using the Cox regression model. *P*-values of < 0.05 were considered as independent influence factors.

## Results

### Patient characteristics

The clinicopathological characteristics of the 140 ESCC patients are summarized in Table 1. According to the 8th edition of the TNM classification of the American Joint Committee on Cancer (AJCC) [26], 74 patients were classified into Stage II and Stage III, and 66 cases were Stage IV. All the patients received the same regimen of concurrent CRT described above. During the study period, CR and non-CR were achieved in 36 and 104 patients, respectively. The CR rate was 25.7%. Among the 104 patients who did not achieve CR, 72 cases received adjuvant chemotherapy, and 10 cases received surgical esophagectomy. The other patients did not receive any antitumor treatments until tumor progression.

**Table 1:** Clinicopathological correlation of DAB2IP expression in ESCC

DAB2IP expression			
Variables	High	Low	*P* value^a^
Age (years)			0.759
≤ 55^b^	22 (24.2%)	69 (75.8%)	
> 55	13 (26.5%)	36 (73.5%)	
Sex			0.187
Male	19 (21.3%)	70 (78.7%)	
Female	16 (31.4%)	35 (68.6%)	
Location			0.399
Cervical/upper thoracic	9 (31.0%)	20 (69.0%)	
Middle/lower thoracic	26 (23.4%)	85 (76.6%)	
WHO grade			< 0.001
G1	21 (61.8%)	13 (38.2%)	
G2	9 (12.3%)	64 (87.7%)	
G3	5 (15.2%)	28 (84.8%)	
Tumor size (cm)			< 0.001
≤ 5^c^	23 (41.8%)	32 (58.2%)	
> 5	12 (14.1%)	73 (85.9%)	
T status			0.003
T2/3	11 (14.9%)	63 (85.1%)	
T4	24 (36.4%)	42 (63.6%)	
N status			< 0.001
N0	20 (62.5%)	12 (37.5%)	
N1	15 (13.9%)	93 (86.1%)	
M status			0.017
M0	27 (32.1%)	57 (67.9%)	
M1-lym	8 (14.3%)	48 (85.7%)	
CRT response			< 0.001
CR	18 (50.0%)	18 (50.0%)	
Non-CR	17 (16.3%)	87 (83.7%)	
Locoregional progression			0.049
Present	10 (16.7%)	50 (83.3%)	
Absent	25 (31.2%)	55 (68.8%)	

*T* tumor, *N* node, *M* metastases, *CRT* chemoradiotherapy, *CR* complete response, *M1-lym* distant lymph node metastasis, *ESCC* esophageal squamous cell carcinoma

^a^Chi-square test

^b^Mean age

^c^Mean tumor size

### Selection of cutoff score for high expression of DAB2IP

**Fig. 1:**
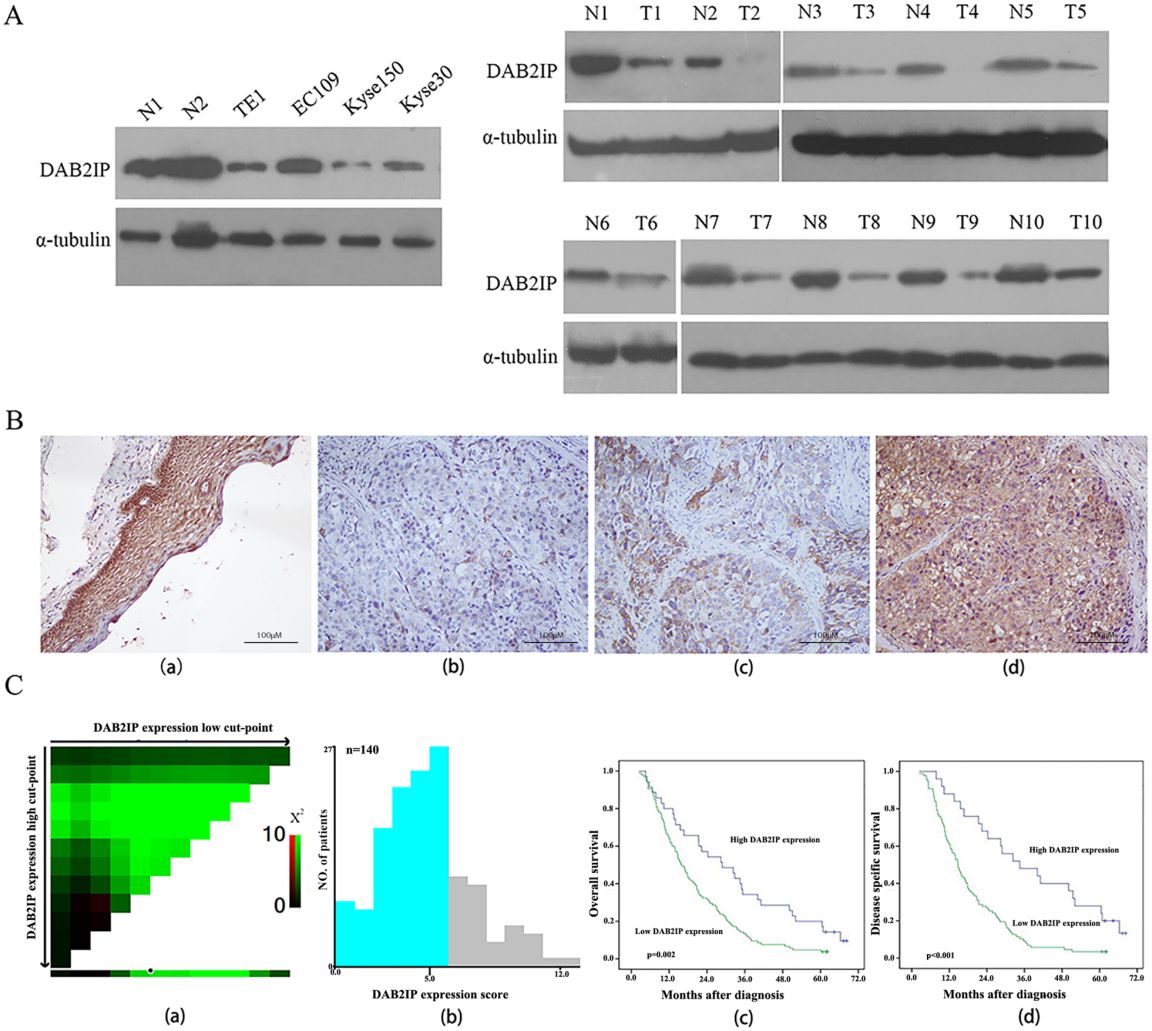
Expression of DAB2IP in ESCC cell lines and tissues and its prognostic significance in ESCC patients. **A** The levels of DAB2IP protein in four ESCC cell lines and normal esophageal mucosa (N1 and N2) examined by Western blot (left); Western blot analysis of DAB2IP protein expression in the 10representative paired of ESCC (T) and normal esophageal mucosa (N) (right). **B** (a) Representative images showed strong DAB2IP IHC signaling of normal esophageal mucosa. (b) Low DAB2IP expression was detected in ESCC case #32 (total score = 1 + 1), with 15% (staining percentage score = 1) carcinoma cells demonstrated weak staining (staining intensity score = 1) of DAB2IP. (c) Moderate DAB2IP expression (total score = 6 + 2) in ESCC case #45, where about 60%-70% (staining percentage score = 6) carcinoma cells demonstrated moderate staining (staining intensity score = 2) of DAB2IP. (d) High expression (total score = 9 + 3) of DAB2IP was observed in ESCC case #91, with 100% (staining percentage score = 9) carcinoma cells demonstrated strong staining (staining intensity score = 3) of DAB2IP. **C** X-tile plots of the prognostic marker DAB2IP. X-tile analysis was conducted on patient data from our ESCC cohort. The plot showed the χ^2^ log-rank values created when the cohort was divided into two population. (a) The cut point highlighted by the black/white circle was demonstrated on the histogram. (b) DAB2IP expression was divided at the optimal cut point, as defined by the most significant on the plot. (c, d) Kaplan–Meier survival analysis of DAB2IP expression for OS (n = 140, (c)) and DSS (n = 112, (d)) in ESCC patients treated with definitive CRT (log-rank test)

To assess statistical significance and avoid the problems of multiple cut-point selection, X-tile program [22] was used to determine an optimal cutoff value for expression of DAB2IP protein while correcting for the use of minimum P statistics by Miller–Siegmund *P*-value correction. The calculated staining score of immunopositive cells ranged from 0 to 12 in all tissues. Based on the cut-point value determined by X-tile software related to survival status, we categorized the samples into low (IHC score ≤ 5) and high (IHC score > 5) expression subgroups (Fig. 1C-a, b).

### Reduced expression of DAB2IP protein in ESCC cell lines and ESCC biopsy tissues

In this study, the protein levels of DAB2IP were examined by Western blotting (Fig. 1A). All the four ESCC cell lines expressed relatively lower levels of DAB2IP than non-cancerous esophageal control tissues. In addition, EC109 cells displayed the highest levels of endogenous DAB2IP expression, whereas Kyse150 cell line showed the lowest level expression (Fig. 1A, left panel). In 14 paired primary tissues, 10/14 (71.4%) showed downregulated DAB2IP expression when compared with adjacent non-neoplastic esophageal tissues. The 10 representative ESCC cases with downregulated expression of DAB2IP are illustrated in Fig. 1A, right panels.

Next, we performed IHC to evaluate the expression of DAB2IP in the 140 ESCC biopsy specimens and 25 corresponding normal mucosal tissues. The DAB2IP staining showed weak and diffuse immunostaining in background stromal cells. Positive signals of DAB2IP protein were predominantly located in the cytoplasm of esophageal cell and also weakly stained in the nucleus. According to the criteria mentioned above, low expression of DAB2IP was observed in 105/140 (75.0%) of primary ESCCs biopsy tissues compared with only 2/25 (8%) in normal esophageal mucosa (Table 1).

### Correlation of DAB2IP expression with clinicopathologic variables

Table 1 summarizes the detailed information about the rates of low expression of DAB2IP with respect to several standard clinicopathological features in the cohort. Results showed that no significant association was found between low DAB2IP expression and some of the clinicopathological features, such as patient’s age, gender, and tumor location (*P* > 0.05). Interestingly, DAB2IP expression correlated closely with World Health Organization (WHO) grade (*P* < 0.001), Tumor size (*P* < 0.001), T status (*P* = 0.003), Node (N) status (*P* < 0.001), and distant lymph node metastasis (M) status (*P* = 0.017) (Table 1).

### Relationship between DAB2IP expression and CRT response

Primary CR was achieved in 25.7% (36/140) of the patients with ESCC. Moreover, using the optimal cutoff value of > 5 score (Fig. 1C-b), DAB2IP expression was also the factor that showed a significant correlation with CRT response in the cohort (r_p_ = 0.322, n = 140, *P* < 0.001) in which low expression of DAB2IP was observed more frequently in the non-CR subset than the CR subset.

### Correlation between clinicopathological variables, DAB2IP expression and ESCC patient survival

Of the 140 patients with ESCC, 8 patients were lost to follow-up. The median survival time was 17.8 months. The 2-year OS and DSS for the entire cohort of patients were 38.6% and 36.6%, respectively. Kaplan–Meier analysis revealed that low-level expression of DAB2IP was associated with short overall survival time (*P* = 0.002) and disease specific survival time (*P* < 0.001) (Fig. 1C-c, d).

In log-rank test, low DAB2IP expression was also found to correlate closely with poor DSS of ESCC patients (median 14.77 vs. 34.50 months, *P* < 0.001; Table 2). Further univariate Cox regression analysis identified WHO grade, tumor size, T status, N status, M status, CRT response and DAB2IP expression to have a significant impact on DSS (*P* = 0.012, 0.003, 0.001, 0.030, 0.022, < 0.001 and < 0.001 respectively; Table 3). Other clinicopathological variables, including age, gender and location showed no significant correlation with DSS (*P* = 0.908, 0.317 and 0.387 respectively; Table 3). The parameters that were significant in univariate analysis were further evaluated in Cox regression multivariate analysis. The results showed that the expression of DAB2IP (*P* < 0.001), T status (*P* < 0.001), and CRT response (*P* = 0.004) were independent predictors of tumor prognosis (Table 4).

**Table 2:** Predictive variables for OS and DSS of ESCC patients by log rank test

Variables	OS (months)			DSS (months)		
	Case	Median	*P*	Case	Median	*P*^a^
Age (years)			0.830			0.908
≤ 55^b^	91	17.10		71	15.40	
> 55	49	17.80		41	17.80	
Gender			0.348			0.316
Male	89	17.80		69	16.70	
Female	51	17.10		43	17.10	
Location			0.353			0.385
Cervical/upper thoracic	29	18.03		24	17.80	
Middle/lower thoracic	111	17.30		88	15.90	
WHO grade			0.069			0.013
G1	34	21.40		26	22.40	
G2	73	18.03		57	15.10	
G3	33	15.70		29	15.50	
Tumor size (cm)			0.004			0.003
≤ 5^c^	55	27.10		41	28.40	
> 5	85	14.77		71	14.60	
T status			< 0.001			0.001
T2/3	74	26.80		64	22.60	
T4	66	13.70		48	12.80	
N status			0.043			0.028
N0	32	22.30		26	22.60	
N1	108	16.90		86	15.10	
M status			0.059			0.020
M0	84	19.90		63	19.30	
M1-lym	56	15.40		49	14.80	
CRT response			< 0.001			< 0.001
CR	36	36.90		31	36.90	
Non-CR	104	14.60		81	13.90	
DAB2IP expression			0.002			< 0.001
High	35	28.80		25	34.50	
Low	105	15.90		87	14.77	

*OS* overall survival, *DSS* disease specific survival

^a^Log-rank test

^b^Mean age

^c^Mean tumor size

**Table 3:** Results of univariate Cox proportional-hazards analysis for DSS for ESCC

Variable	*P*-values	Hazard ratios	95% CI
Age (years)	0.908	0.977	0.654–1.458
Gender	0.317	0.814	0.544–1.218
Location	0.387	0.813	0.509–1.299
WHO grade	0.012	1.407	1.077–1.839
Tumor size (cm)	0.003	1.839	1.224–2.764
T status	0.001	1.929	1.306–2.849
N status	0.030	1.670	1.051–2.654
M status	0.022	1.585	1.070–2.348
CRT response	< 0.001	4.569	2.752–7.585
DAB2IP expression	< 0.001	2.836	1.708–4.710

*CI* confidence interval

**Table 4:** Cox multivariate analyses of prognostic factors on disease specific survival

Variable	*P*-values	Hazard ratios (95% CI)
T status	< 0.001	
T2/3		1.000
T4		2.975 (1.869–4.736)
CRT response	0.004	
CR		1.000
Non-CR		2.528 (1.350–4.735)
DAB2IP expression	< 0.001	
High		1.000
Low		4.056 (1.855–8.867)

*CI* confidence interval

### DAB2IP regulates the chemosensitivity of ESCC cells to cisplatin in vitro

**Fig. 2:**
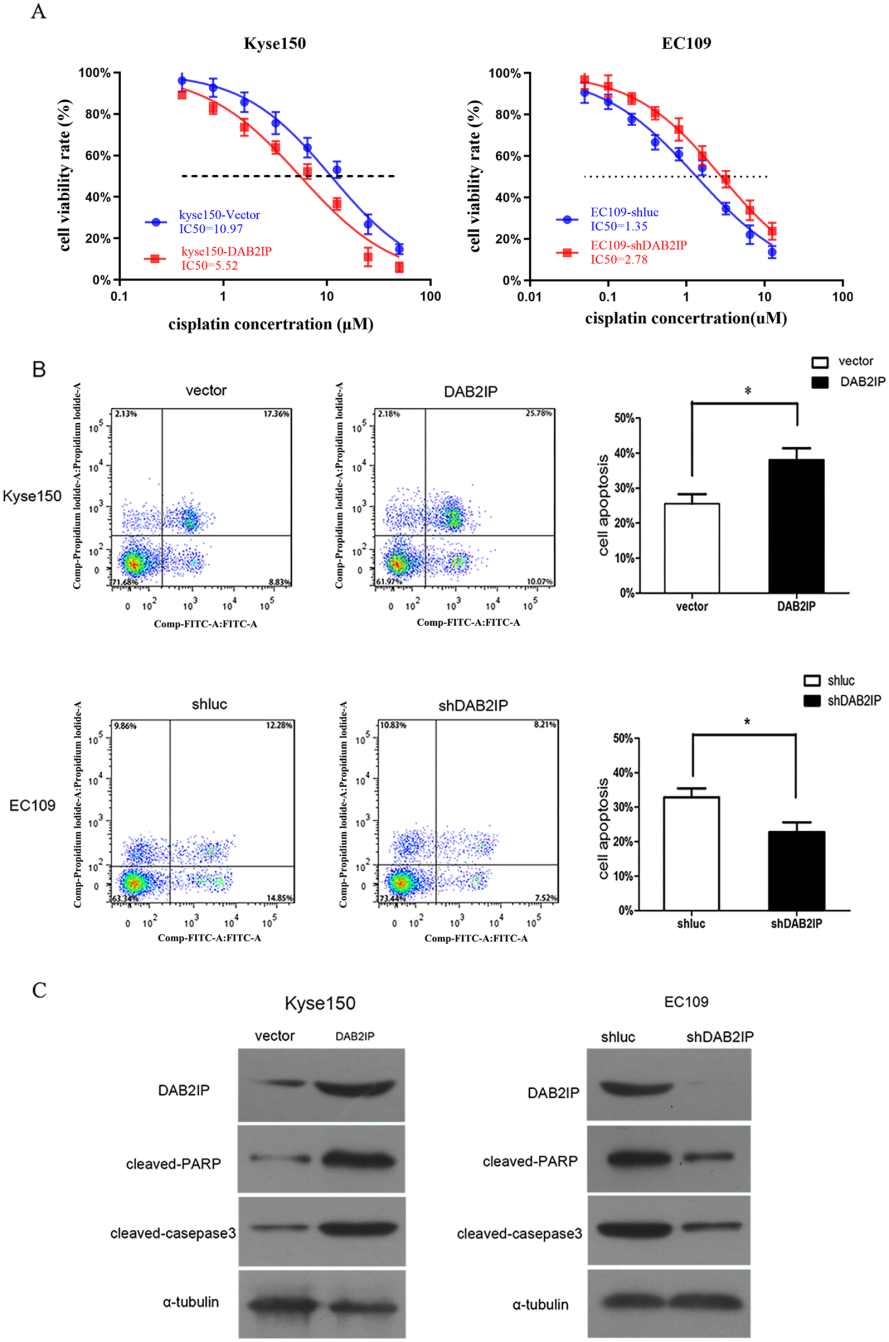
The impact of DAB2IP levels on the chemosensitivity of ESCC cells in vitro. **A** Shift of dose–response curves of cisplatin by overexpression or knockdown of DAB2IP was determined by MTS assay. Overexpression of DAB2IP in Kyse150 cells is significantly more sensitive to cisplatin than parental control cells (left). Consistently, knockdown of DAB2IP reduced sensitivity of EC109 cells to cisplatin (right). IC50 values were shown below. **B** Upper panels, Kyse150-DAB2IP and the control Kyse150-vector cells were treated with cisplatin (IC30), and 24 h later, the cells were collected and stained with PI and AnnexinV. Lower panels, EC109-shluc and EC109-shDAB2IP cells were incubated with cisplatin (IC30). After 24 h, Annexin V and PI staining assay was used to determine the percentage of cells undergoing apoptosis. The p-values were calculated by the Student’s t test. The bar shows the mean ± SD of three independent experiments. *p < 0.05. **C** Left, 24 h after cisplatin (IC30) treatment, overexpression of DAB2IP increased the levels of cleaved PARP and cleaved caspase-3 in Kyse150 cells. Right, silencing of DAB2IP decreased cisplatin-induced levels of cleaved PARP and cleaved caspase-3 in EC109 cells

To further investigate whether DAB2IP is capable of modulating the chemoresistance of ESCC cells in vitro, the Kyse150 cell line, which showed the lowest endogenous expression levels of DAB2IP, was subsequently transfected with pcDNA3.1-DAB2IP or the control plasmid pcDNA3.1. As shown in Fig. 2A left, ectopic expression of DAB2IP in Kyse150 cells (Fig. 2C, left) resulted in a substantial reduction of IC_50_ value under cisplatin treatment compared to that of the empty vector control cells (5.52 µM vs. 10.97 µM). Consistently, stably silencing DAB2IP by lentivirus in EC109 cells, which showed a relative high expression of DAB2IP (Fig. 1A, left panel), resulted in increased IC_50_ value compared with the control EC109-shluc cells (1.35 µM vs. 2.78 µM, Fig. 2A, right). The knockdown efficiency of shRNA lentivirus was validated by Western blot (Fig. 2C, right).

To further identify the crucial role of DAB2IP in ESCCs chemoresistance, the Annexin V/PI assay was performed to evaluate the effect of DAB2IP on apoptosis. As illustrated in Fig. 2B, the proportion of apoptosis was dramatically increased when Kyse150-DAB2IP cells were pretreated with cisplatin (25.560 ± 4.680% vs. 38.160 ± 5.620%, *P* = 0.041, Fig. 2B, upper panels). Accordantly, ablation of endogenous DAB2IP remarkably reduced cisplatin-induced apoptosis in EC109 cells (32.840 ± 4.57% vs. 21.280 ± 4.890%, *p* = 0.0403, Fig. 2B, lower panels).

In agreement with the results obtained by Annexin V/PI assay, our following Western blot analysis indicated that the ectopic expression of DAB2IP in Kyse150 cells obviously increased protein levels of cleaved-caspase 3 and cleaved-PARP induced by cisplatin (Fig. 2C, left). On the other hand, the depletion of endogenous DAB2IP in EC109 cells led to a substantial reduction of cleaved-caspase 3 and cleaved-PARP in the presence of cisplatin (Fig. 2C, right**)**. Taken together, these results provide strong evidence that DAB2IP plays a crucial role in regulating chemosensitivity of ESCC cells.

### DAB2IP influences the sensitivity of ESCC cells to ionizing radiation (IR) in vitro

**Fig. 3:**
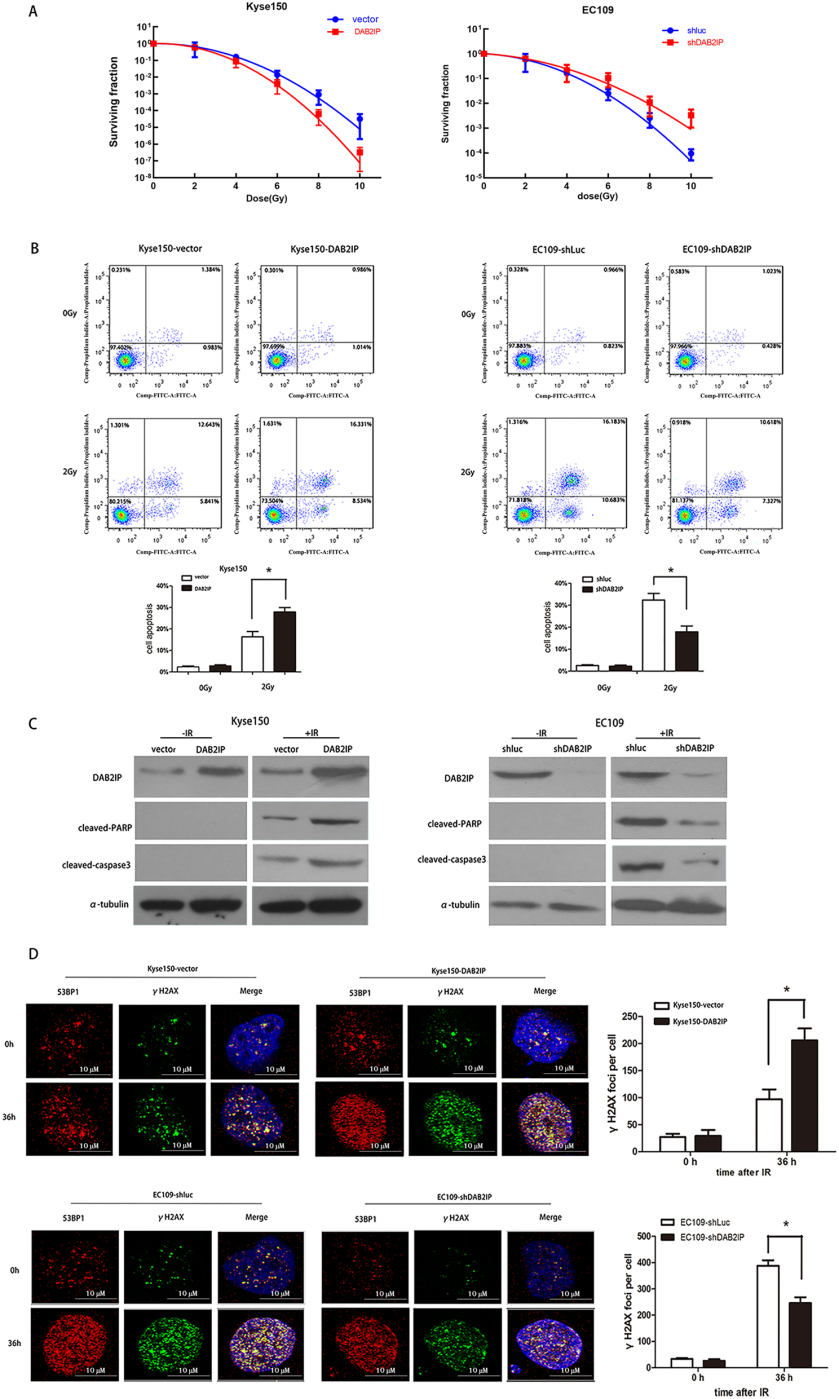
The expression levels of DAB2IP modulate the radiosensitivity of ESCC cells in vitro. **A** Left, clonogenic survivals of DAB2IP overexpressed cells of Kyse150 cell lines were reduced compared to parental cells after a single dose radiation (0, 2, 4, 6, 8 and 10 Gy). Right, knockdown of DAB2IP increased clonogenic survival fraction of EC109. All data were from three independent experiments, and were fitted according to the linear quadratic mode. **B** Left panels, Kyse150-DAB2IP and control Kyse150-vector cells were treated with or without IR (3 Gy), and 48 h later, the cell apoptotic death events were monitored by Annexin V/PI staining cells. Right panels, EC109 cells, initially infected with DAB2IP-shRNA and Luc-shRNA, were treated with or without IR. After 48 h, Annexin V/PI staining was used to determine the percentage of cells undergoing apoptosis. All data described are the mean ± SD of three independent experiments. *p < 0.05. All data are reported as mean ± SD. **C** Forty-eight hours after the cells were treated with IR (3 Gy), the cleavages PARP and cleaved casepase-3 were detected in Kyse150-vector/Kyse150-DAB2IP (left) and EC109-shluc/EC109-shDAB2IP (right) cells by Western blot analysis. Experiments were performed three times and a representative result is shown. **D** The levels of DAB2IP affect IR-induced DNA damage in ESCC cells. Kyse150-vector/Kyse150-DAB2IP (upper panels) and EC109-shluc/EC109-shDAB2IP (lower panels) were irradiated with 3 Gy and immunostained for 53BP1 (red) and phospho-γH2AX (green) foci at the indicated time points after radiation. Colocalized foci (yellow) were used as a measure of DSB. Nuclei were counterstained with DAPI (blue). Columns (right) indicate quantification of average numbers of IR-induced colocalized foci per cell (0 or 36 h after IR). A total of 100 cells from three independent experiments were counted for each group. The data represent mean ± SD. (*p < 0.05, p-value was according to Student’s t test)

Since our above ESCC cohort data revealed that DAB2IP expression was significantly correlated with CRT response (Table 1), we next used clonogenic assay to determine the important role of DAB2IP in ESCC radiosensitivity in vitro. As shown in Fig. 3A, ectopic overexpression of DAB2IP in Kyse150 cells resulted in an obviously reduction of clonogenic survival fractions when compared with the corresponding control Kyse150-vector cells (Fig. 3A, left). In addition, increment of clonogenic survival fractions was observed in DAB2IP repressed EC109 cells in comparison to control EC109-shluc cells. Additional non-linear curve-fitting analysis of the dose–response survival curves was performed according to the linear quadratic (LQ) model, and the parameters for each curve including α value, β value, α/β value, SF_2_, and sensitizing enhancement ratio (SER) were listed (Table 5). According to these values, SF_2_ was markedly reduced in Kyse150-DAB2IP cells compared to the control Kyse150-vecter cells (0.43 vs. 0.66) with consequence that SER value was 1.52. Consistently, a substantial increase of SF_2_ value was observed in DAB2IP-silenced EC109 cells when compared with control EC109-shluc cells (0.67 vs. 0.59), which indicated that the SER of DAB2IP silencing in EC109 cells was 0.87 (Table 5).

**Table 5:** Clonogenic survivals were fitted to the linear quadratic model and SER was calculated

	α value (Gy^−1^)	β value (Gy^−2^)	α/β value (Gy)	SF_2_	SER (ratio of SF_2_)
Kyse150-vector	− 0.03	0.12	− 0.25	0.66	1.52
Kyse150-DAB2IP	0.08	0.17	0.47	0.43	
EC109-shLuc	0.09	0.09	0.95	0.59	0.87
EC109-shDAB2IP	0.05	0.07	0.68	0.67	

SF_2_: survival fraction at 2 Gy; SER: sensitizing enhancement ratio

Next, to obtain more evidence regarding the involvement of DAB2IP protein in regulating ESCC cell radiosensitivity, Annexin V/PI assay and immunoblots for detecting the apoptosis-related marker proteins (i.e., cleaved caspase-3 and cleaved PARP) was performed. As shown in Fig. 3B, C, with the absence of IR, neither overexpression nor knockdown of DAB2IP could influence the proportion of apoptosis and the levels of apoptosis-related marker proteins. This phenomenon had changed after treating cells with 3 Gy X-rays. In the presence of IR, both Annexin V/PI assay and Western blot results indicated that the apoptotic activity of DAB2IP was significantly enhanced in DAB2IP-overexpressed Kyse150 cells in contrast to control Kyse150-vector cells, whereas repression DAB2IP in EC109 cells cause a remarkable decrease of apoptotic cells (32.360 ± 5.210% vs. 17.890 ± 4.65%, *P* = 0.023), cleaved caspase-3, and cleaved PARP when compared with corresponding control EC109-shLuc cells.

### The radiosensitizing activity of DAB2IP is associated with enhanced double-stranded DNA breaks (DSB) repair in ESCC cells

It is wellknown that the DSB repair capacity is closely correlated with intrinsic radiosensitivity of cells [27]. DSB repair involves the rapidly phosphorylation of γH2AX and the P53 binding protein (53BP1) recruitment in site-specific DNA damage [28]. Thus, the formation of γ-H2AX and 53BP1 foci, which displayed as discrete foci at surrounding DNA double-strand breaks, is widely used to monitor radiation-induced DNA breaks and to assay DNA rejoining defects [29].

To investigate whether DAB2IP can influence DNA repair ability of ESCC cells, the dual immunofluorescence staining for 53BP1 (red) and phospho-γH2AX (green) foci was performed. The DSB repair kinetics was determined by counting the colocalized foci (yellow). As illustrated in Fig. 3D upper panels, after 36 h of IR treatment, the unrepaired DNA damage detected by counting colocalized foci of γH2AX and 53BP1 was significantly increased in Kyse150-DAB2IP cells when compared with control Kyse150-vector cells (97 ± 18 vs. 206 ± 22, *P* = 0.003), while no obvious difference was observed before IR treatment (0 h). Consistently, 36 h after IR exposure, the colocalized foci number of γH2AX and 53BP1 was remarkably reduced in DAB2IP-silenced EC109 cells when compared to control EC109-shluc cells (388 ± 36 vs. 246 ± 38, *p* = 0.038). However, without IR treatment (0 h), no distinct difference was detected in these two group cells. These data indicated that the expression of DAB2IP was associated with DSB repair capability of ESCC cells.

### Overexpression of DAB2IP enhances the radiosensitivity of esophageal squamous cell carcinoma cells in vivo

**Fig. 4:**
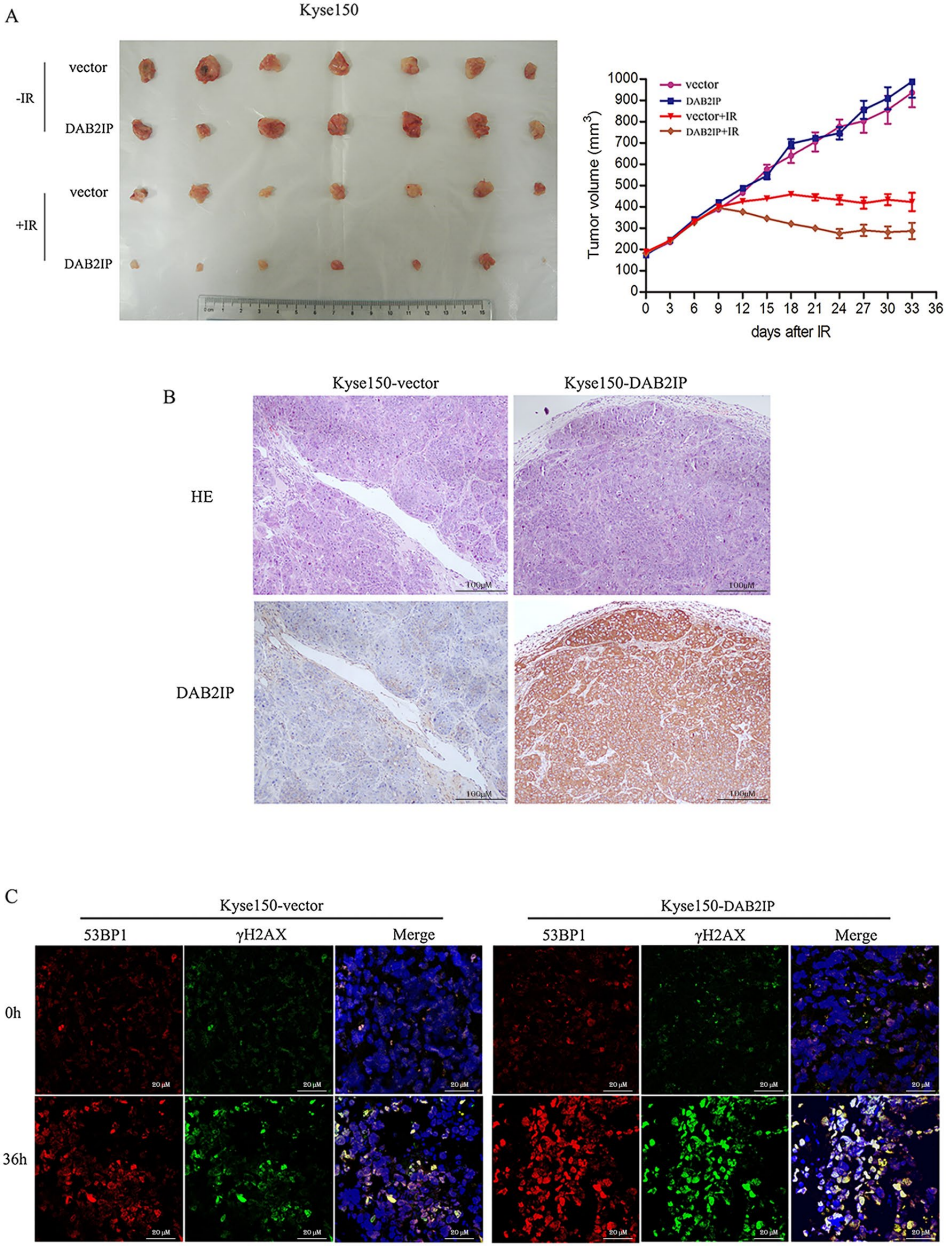
Upregulated expression of DAB2IP enhances the killing-effect of IR on ESCC cell xenografts. **A** Kyse150-DAB2IP or control Kyse150-vectorcells (106) were injected subcutaneously into right flank of athymic nude mice, respectively. When the volume of a transplanted tumor grew to 180 mm^3^, the tumors were received 6 Gy dose of IR. The tumor volume of xenografts was measured with calipers every 3 days for a total of 33 days. Overexpressed DAB2IP alone did not affect the growth of the tumors; the mean tumor volume in the Kyse150-vector and Kyse150-DAB2IP groups was 936 ± 68 mm^3^ and 989 ± 76 mm^3^, respectively (n = 7, p = 0.1943, Student’s t test). However, when treated transplanted tumor with 6 Gy dose of IR, the mean tumor volume of the Kyse150-vectorgroup was significantly larger than that of the Kyse150-DAB2IP group (423 ± 43 mm^3^ vs. 287 ± 38 mm^3^, p ≤ 0.05). The values represent mean tumor volume ± SD. **B** Representative images of DAB2IP IHC staining in Kyse150-vector and Kyse150-DAB2IP transplanted tumor tissues. **C** Nude mice xenograft from Kyse150-vector and Kyse150-DAB2IP cells were treated with 6 Gy dose of IR, and 0 or 24 h later, immunofluorescence with antibodies with γH2AX (green) and 53BP1 (red) was performed. Merged spots (yellow) show colocalization of 53BP1 and γH2AX foci at DSBs. Experiments were performed three times and a representative result is shown

To further determine whether DAB2IP has similar impact on ESCC cell response to IR in vivo, Kyse150-DAB2IP and control Kyse150-vector cells were inoculated into female BALB/c nude mice. In agreement with our in vitro experiments, we found that overexpression of DAB2IP alone did not influence tumorigenicity of ESCC cells, (i.e., both the injected Kyse150-DAB2IP and control Kyse150-vector cells showed a similar efficiency and growth rate of the ESCC xenografts in nude mice) (Fig. 4A). However, when the mice-bearing tumors received 6 Gy of IR, the growth of the Kyse150-DAB2IP tumors was significantly inhibited (from a mean tumor volume of 180 mm^3^ before IR treatment, increasing to 287.00 ± 38.00 mm^3^ at the endpoint of observation). In comparison, the increase in tumor volume of control Kyse150-vector was greater, from a mean tumor volume of 180 mm^3^ to 423.00 ± 43.00 mm^3^) (*P* < 0.01; Fig. 4A).

The stably overexpressed levels of DAB2IP measured by IHC staining in Kyse150 xenograft tumors are shown in Fig. 4B. Similar to the results observed in above in vitro experiment, the in vivo radiation-induced γH2AX foci assay indicated that, 24 h after receiving 6 Gy radiation, the unrepaired DNA damage was substantially increased in Kyse150-DAB2IP xenograft tumor tissues compared to parental control cell line xenograft tumors (Fig. 4C).

Collectively, the in vivo and in vitro data demonstrated that DAB2IP plays a crucial role in modulating radiosensitivity of ESCC cells.

### DAB2IP regulates ESCC cell radiosensitivity, possibly through enhancing IR-induced activation of ASK1-JNK signaling

**Fig. 5:**
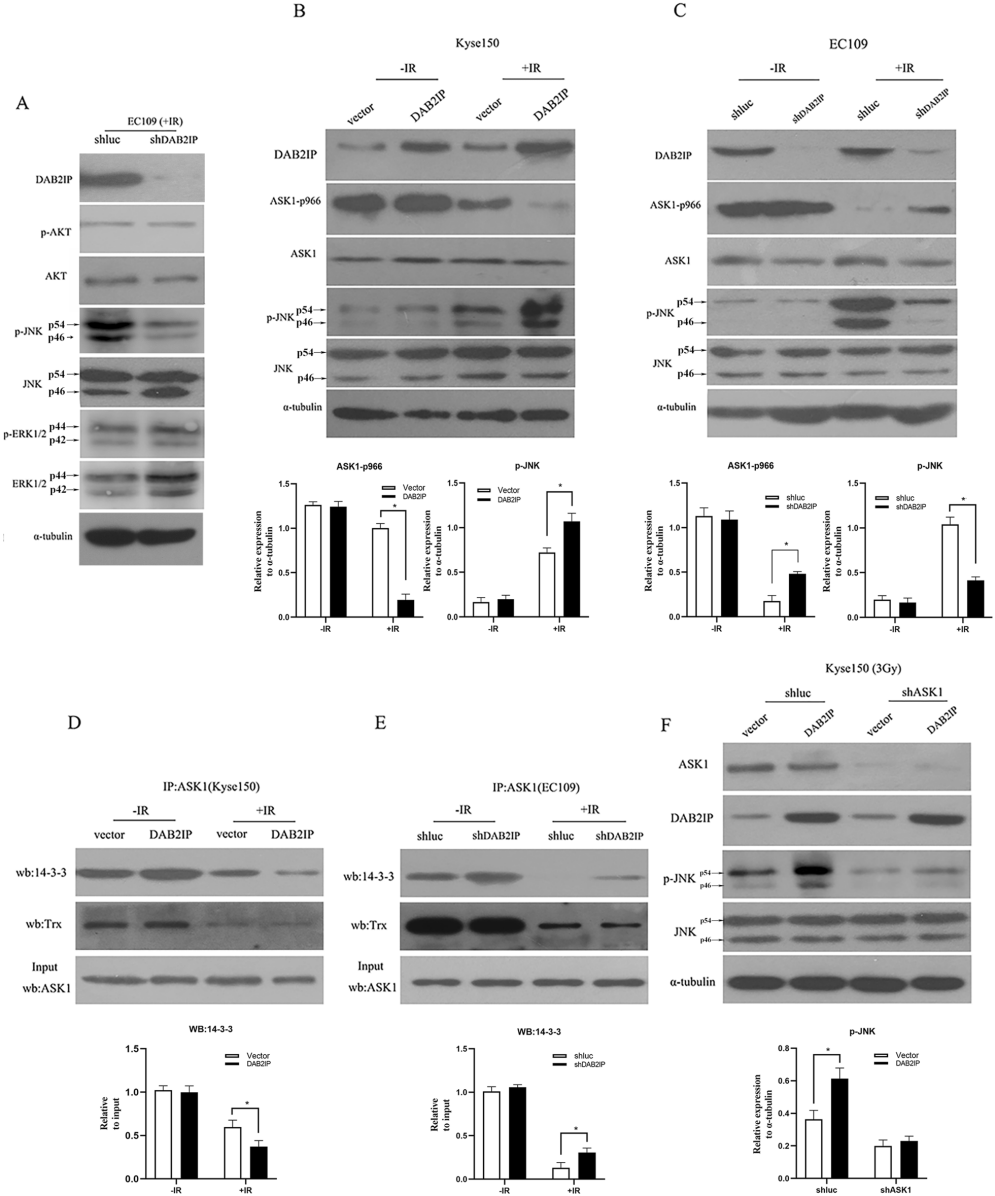
The critical role of DAB2IP in modulating IR-induced ASK1-JNK pathway activation. **A** EC109-shluc and EC109-shDAB2IP cells were initially treated with 3 Gy dose of IR, and 36 h later, the indicated protein levels were evaluated by Western blotting. Silencing DAB2IP by lentivirus obviously increased the protein levels of p-JNK. No changes were observed in p-ERK and p-AKT levels. **B** DAB2IP enhanced IR-induced dephosphorylation of ASK1-pSer966 and JNK activation. Kyse150 cells were initially transfected with pcDNA3.1-DAB2IP or vehicle pcDNA3.1 plasmid, followed by treatment with or without IR, and 36 h later, ASK1-pSer966, and JNK activation were measured by Western blotting. **C** EC109 cells were firstly infected with DAB2IP-shRNA or control luc-shRNA lentivirus, and subsequently received with or without IR. After 36 h, Western blotting was performed to detect levels of ASK1-pSer966 and p-JNK. Results are representative data of three independent experiments. **D** DAB2IP enhanced IR-induced disruption of ASK1-14-3-3 complex, but not ASK1-Trx complex. Kyse150 cells were initially transfected with pcDNA3.1-DAB2IP or vehicle pcDNA3.1plasmid, followed by treatment with or without IR, and 36 h later, cell extracts were firstly immunoprecipitated by anti-ASK1 antibody, and then the precipitate was immunoblotted (IB) with 14-3-3 and Trx antibody. Input was equivalent to 30% of the cell lysate used in the immunoprecipitation. **E** EC109 cells were initially infected with lentivirus carried with DAB2IP-shRNA or luc-shRNA, subsequently received with or without IR. After 36 h, interaction of ASK1 with endogenous 14-3-3 or Trx was determined by coimmunoprecipitation with primary anti-ASK1 antibody followed by Western blot with 14-3-3 or Trx antibody. The input represents 30% of the cell lysates. Each immunoprecipitation experiment was repeated at least three times and a representative experiment is shown. **F** The enhanced activity of DAB2IP on IR-activated JNK signal was dependent on the presence of ASK1. The Kyse150 cells were firstly infected with ASK1-shRNA or control luc-shRNA lentivirus, and then each group cells were transfected with pcDNA3.1-DAB2IP or vehicle pcDNA3.1 plasmid. All group cells were followed by 3 Gy IR treatment, and 36 h later, JNK activation were evaluated by Western blotting with p-JNK antibody. Arrows point to the bands representing indicated proteins. The representative blots of three independent experiments were shown

Apoptosis is an important mechanism by which IR exerts its therapeutic response and faulty apoptosis is a known mechanism leading to resistance to radiation therapy [30]. In previous literature, DAB2IP has been demonstrated to be involved in several apoptotic pathways, including Ras/Raf/MEK/ERK (MAPK) signaling, PI3K/Akt signaling and ASK1-JNK signaling [24, 31, 32]. Therefore, to address which signaling pathways were critically involved in DAB2IP regulating ESCC cells radiosensitivity, we initially performed Western blot to evaluate the phosphorylation levels of AKT, JNK, and ERK, which represent the activated degree of these three signaling pathways, respectively. As shown in Fig. 5A, in the present of 3 Gy IR treatment, stably silencing DAB2IP by lentivirus in EC109 cells obviously increased the protein levels of p-JNK. No changes were observed in p-ERK and p-AKT levels. This result implied that ASK1-JNK signaling pathway might be an important mechanism in DAB2IP regulating ESCC cells radiosensitivity.

### DAB2IP facilitates IR-induced dephosphorylation at Ser-966 of ASK1 accompanied by reinforced phosphorylation of JNK

Next, to confirm whether ASK1-JNK pathway was involved in DAB2IP-inducedregulation of ESCC cells radiosensitivity, the stably ectopic DAB2IP-overexpressed Kyse150 cells were established and followed by 3 Gy X-ray irradiation. The dephosphorylated levels of ASK1 at Ser966 and phosphorylated levels of JNK, both triggered by X-ray irradiation, were determined by Western blot. As illustrated in Fig. 5B, elevated expression of DAB2IP alone in Kyse150 cells did not alter p-ASK1ser966 and p-JNK levels in the absence of IR treatment. However, when treated cells with 3 Gy IR, ectopic overexpression of DAB2IP remarkably reduced the levels of p-ASK1-ser966, concomitant with a substantially increased levels of p-JNK. These observations were further supported by our results from knockdown experiments. As anticipated, silencing DAB2IP by lentivirus alone in EC109 cells did not affect the protein levels of ASK1 p-Ser966 and p-JNK in the condition without IR. However, in the presence of IR, we observed dramatically increased levels of p-ASK1 Ser966 concurrently with virtual reduction of p-JNK in DAB2IP-depleted EC109 cells when compared with our control EC109-shluc cells (Fig. 5C).

### DAB2IP enhances IR-induced ASK1 activation by facilitating dissociation of ASK1 from its inhibitor 14-3-3 but not from its other inhibitor, thioredoxin (Trx)

It has been well documented that 14-3-3 is one of the most important inhibitors of ASK1. The dephosphorylation at Ser-966 of human ASK1 and consequent dissociation of 14-3-3 with ASK1 has been thought to be a crucial activation mechanism of ASK1-JNK pathway [33–36]. In addition, it was further reported that DAB2IP could enhance TNF-α-induced ASK1 activation by facilitating dissociation of inhibitor 14-3-3 from ASK1 [37]. Thus, we posited that DAB2IP might be capable of intensifying IR-induced ASK1 activation through a similar mechanism. To test this premise, Kyse150 cells were firstly transfected with vector pcDNA3.1 or pcDNA3.1-DAB2IP plasmid, then cells were treated with or without IR, and the association of ASK1 with 14-3-3 was determined by immunoprecipitation with anti-ASK1 followed by Western blot with anti-14-3-3. As expected, the interaction between ASK1 and 14-3-3 was substantially reduced in response to IR treatment, whereas this reduction was reinforced by elevated expression of DAB2IP (Fig. 5D). Accordingly, the radiation-induced disruptive effect on the ASK1-14-3-3 complex was also observed in EC109 cells, and this disruptive effect was markedly attenuated by knocking down of DAB2IP (Fig. 5E).

Besides, we also determined whether DAB2IP could affect IR-induced dissociation of ASK1 with Trx, another important negative regulator of ASK1. Intriguingly, our coimmunoprecipitation assay showed that neither knockdown nor overexpression of DAB2IP could impact on IR-induced disruption of ASK1-Trx complex (Fig. 5D, E). This important result has excluded the possibility that DAB2IP regulated IR-induced ASK1 activation by enhancing the dissociation of ASK1 with Trx.

### The enhanced activity of DAB2IP on IR-activated JNK signals dependent on the presence of ASK1

In order to further identify whether the sensitizing effect of DAB2IP in response to IR was through ASK1-JNK pathway, Kyse150 cells were firstly stably knocked down ASK1 by lentivirus, and subsequently, the control pcDNA3.1 or pcDNA3.1-DAB2IP plasmid was transfected into these cells to determine whether DAB2IP can still activate JNK signal in the absence of ASK1. As shown in Fig. 5F, in the presence of IR, the enhanced activity of DAB2IP toward JNK signal was virtually totally prevented in ASK1-depleted Kyse150 cells. These data suggested that the presence of ASK1 was required and might be a predominant mediator for the enhanced activity of DAB2IP toward JNK signal.

## Discussion

In our study, we found that decreased expression of DAB2IP in ESCC patients treated with definitive chemoradiotherapy correlated positively with ESCC resistance to CRT and was a strong and independent predictor for short disease-specific survival (DSS) of ESCC patients. Subsequently, the crucial role of DAB2IP in regulating ESCC chemoradiosensitivity was confirmed by a series of in vitro and in vivo experiments. Significantly, our Western blot results showed that p-JNK was reduced in DAB2IP-silenced EC109 cells, suggesting that the ASK1-JNK pathway might be a crucial mechanism involved in DAB2IP modulation of the radiosensitivity of ESCC cells.

Apoptosis signal-regulating kinase 1 (ASK1) was initially identified as a MAP3K, and it selectively activates the c-Jun N-terminal kinase (JNK) and p38 pathways in response to various stimuli [38]. One of the most important activators of ASK1 is oxidative stress. Under normal physiological conditions, ASK1 is maintained in an inactive state through binding to the inhibitory factors thioredoxin (Trx) and 14-3-3 proteins [33, 39]. In response to ROS stimulation, Trx is oxidized and dissociated from ASK1. This ROS-induced dissociation of Trx may initiate a conformational change in ASK1, exposing phosphorylated serine residue site (Ser-966 in humans) to a bound serine phosphatase. The phosphorylation of Ser-966 causes the release of 14-3-3 proteins from ASK1, which might finally lead to ASK1 activation [36, 40].

To elucidate the detailed mechanism of the ASK1-JNK pathway involvement in DAB2IP regulation of ESCC radiosensitivity, a series of experiments were conducted. Unexpectedly, our Western blot results indicated that ectopic overexpression of DAB2IP alone did not induce the dephosphorylation of Ser-966, and release of 14-3-3 from ASK1, as well as activation of JNK pathways. However, when cells were treated with 3 Gy IR, overexpression of DAB2IP obviously enhanced the IR-induced dephosphorylation of Ser-966, leading to increased dissociation of ASK1 from 14-3-3 and the subsequent augmented activation of the JNK pathway. These results suggested that IR might remove the inhibitory factors that are required for the enhanced activation function of DAB2IP on ASK1-JNK signaling.

It is well established that IR can either directly or indirectly generate intracellular reactive oxygen species (ROS), which function as a key intermediates in the radiation mediated induction of apoptotic signaling in tumor cells [41–43]. As we mentioned above, when Trx is oxidized by ROS, it releases from ASK1, permitting ASK1 to be dephosphorylated on Ser-966 by ROS, which, in turn, induces dissociation of ASK1 from its inhibitor protein 14-3-3, ultimately leading to activation of the ASK1-JNK pathway [33, 36, 39, 40]. Accordingly, our immunoprecipitation results showed that IR indeed triggers dissociation of ASK1 from both 14-3-3 and Trx (Fig. 5D, E). This step is necessary for basic activation of ASK1 but not sufficient for intensive ASK1 activation. It is conceivable that IR induces release of 14-3-3 and Trx in a coordinated fashion, permitting DAB2IP to further intensify ASK1 activation. Notably, we observed that only the dissociation of ASK1 from 14-3-3, but not Trx, can be enhanced by DAB2IP. This result excludes the possibility that DAB2IP enhances IR-induced ASK1 activation by facilitating the dissociation of ASK1 from Trx (Fig. 5D, E).

Additionally, previous important study demonstrated that, with the stimulation of TNF-α, DAB2IP preferentially binds to the locus surrounding the Ser-966-dephosphorylated site of ASK1 and facilitates dissociation of 14-3-3 through recruitment of the okadaic acid-sensitive phosphatase PP2A to the ASK1 complex, which ultimately leads to the enhanced activation of JNK [37, 44, 45]. In agreement with this previous result, our present study also found that DAB2IP could enhance IR-induced dephosphorylation of ASK1-Ser-966, which in turn, facilitates 14-3-3 release from ASK1, ultimately leading to intensive activation of ASK1 (Fig. 5B, D).

**Fig. 6:**
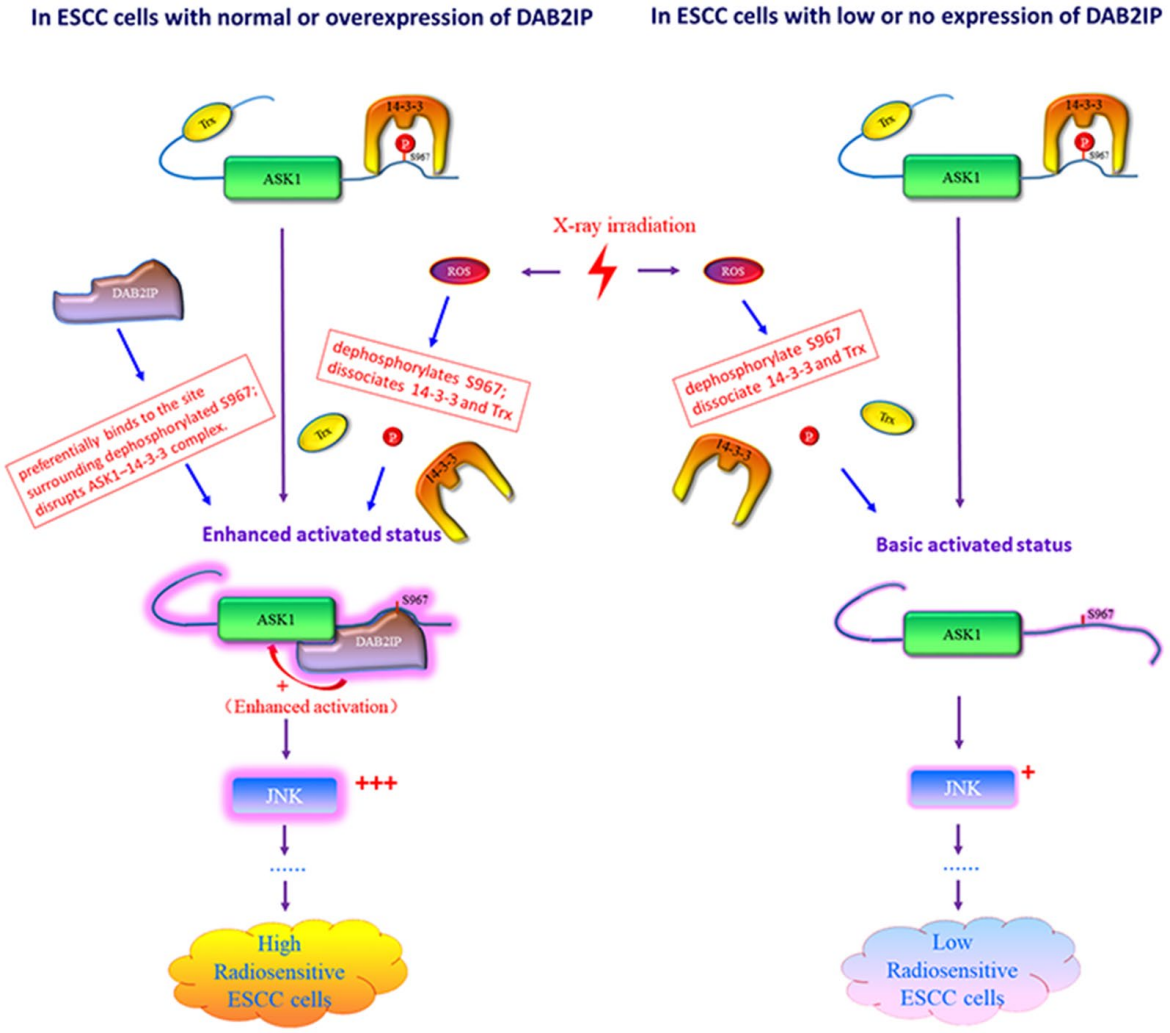
Proposed schematic representation for the molecular mechanism of DAB2IP-inducedregulation of radiosensitivity in ESCC cells. In ESCC cells with low or no expression of DAB2IP, IR causes increased levels of intracellular reactive oxygen species (ROS), leading to the dephosphorylation of Ser-966 of ASK1, which consequently triggers dissociation of Trx and 14-3-3 from ASK1, ultimately resulting in a basic activated status of the ASK1-JNK pathway and causing ESCC cells to exhibit low sensitivity/or resistance to radiotherapy. In ESCC cells with normal or overexpression of DAB2IP, the IR-induced ROS stimulates dephosphorylation of Ser-966 of ASK1, thereby releasing of 14-3-3 and Trx from ASK1. This step is necessary but not sufficient for ASK1-enhanced activation. After 14-3-3 and Trx are released from ASK1, DAB2IP preferentially binds to the locus surrounding the Ser966-dephosphorylated site of ASK1, which consequently facilitates dissociation of 14-3-3 from ASK1 and enhances ASK1 kinase activity, ultimately leading to an over-activation of ASK1-JNK signaling and ESCC cells with high sensitivity to radiotherapy

From our collective data, we propose a possible model to illustrate the mechanism of DAB2IP-mediated regulation of radiosensitivity of ESCC cells in Fig. 6. Based on this model, we can easily explain the functional results observed in Figs. 3 and 4. That is, without IR treatment, the apoptotic activity of ASK1 was completely repressed by its inhibitors Trx and 14-3-3; thus, without IR treatment, overexpressed DAB2IP per se was incapable of inducing cell apoptosis or suppressing the tumorigenicity of ESCC cells in nude mice (Figs. 3B, C and 4A). These results also support the evidence that the release of Trx and 14-3-3 triggered by IR was required for the enhanced killing effects of DAB2IP. Since the mechanisms by which IR kills cells are very complicated and still poorly understood, it is possible that other unknown pathways might also involve in DAB2IP modulation of the radiosensitivity of ESCC cells. Clearly, further studies are needed to confirm this model and elucidate whether other pathways might also contribute to DAB2IP-mediated regulation of the radiosensitivity of ESCC cells.

## Conclusions

In summary, our study described the protein expression of DAB2IP in normal human esophageal tissues and biopsy specimens of primary ESCC patients treated with definitive CRT. In addition, our results provide a basis for the concept that downregulation of the expression DAB2IP, as detected by IHC, may be a useful tool for predicting CRT resistance and an independent prognostic factor of patients with ESCC treated with definitive CRT. We further demonstrated that the sensitizing effect of DAB2IP in response to IR is through enhanced activation of the ASK1-JNK pathway. These findings might be helpful in clinical practice for selecting potential radioresistant ESCC patients who might require a more intensive follow-up and/or more aggressive treatment.

## Data Availability

The datasets supporting the conclusions of this article are included within the article and could be obtained from the corresponding author if necessary.

## Abbreviations

DAB2IP: Disabled homolog 2 interacting protein
ESCC: Esophageal squamous cell carcinoma
IHC: Immunohistochemistry staining
CRT: Chemoradiotherapy
IR: Ionizing radiation
CR: Complete response
OS: Overall survival
DSS: Disease-specific survival
SER: Sensitizing enhancement ratio
53BP1: The P53 binding protein
Trx: Thioredoxin
JNK: Jun N-terminal kinase
ERK: Extracellular regulated protein kinases
